# miR-885-5p suppresses hepatocellular carcinoma metastasis and inhibits Wnt/β-catenin signaling pathway

**DOI:** 10.18632/oncotarget.12602

**Published:** 2016-10-12

**Authors:** Zhuhong Zhang, Jing Yin, Jian Yang, Wenzhi Shen, Chunyan Zhang, Wenjun Mou, Jinhua Luo, Hua Yan, Peiqing Sun, Yunping Luo, Yaping Tian, Rong Xiang

**Affiliations:** ^1^ Department of Immunology, School of Medicine, Nankai University, Tianjin, 300071, China; ^2^ Department of Clinical Biochemistry, Chinese PLA General Hospital, Beijing, 100853, China; ^3^ Department of Ophthalmology, Tianjin Medical University General Hospital, Tianjin, 300052, China; ^4^ The Scripps Research Institute, La Jolla, CA, 92037, USA; ^5^ Department of Immunology, Institute of Basic Medical Science, Chinese Academy of Medical Science and Peking Union Medical College, Beijing, 100005, China; ^6^ Current address: Division of Genetic and Molecular Toxicology, National Center for Toxicological Research, FDA, Jefferson, AR 72079, USA

**Keywords:** miR-885-5p, Wnt/β-catenin HCC, migration, invasion

## Abstract

MicroRNAs (miRNAs) inhibit or improve the malignant progression of hepatocellular carcinoma (HCC). We previously reported that compared to health controls, patients with liver cirrhosis present the highest levels of circulating miR-885-5p, followed by those with chronic hepatitis B and those with HCC. However, the molecular involvement of miR-885-5p in HCC metastasis is presently unclear. Here, we demonstrated that the expression of miR-885-5p negatively correlated with the invasive and metastatic capabilities of human HCC tissue samples and cell lines. We found that miR-885-5p expression levels correlated with the survival of patients with HCC. Overexpression of miR-885-5p decreased metastasis of HCC cells *in vitro* and *in vivo*. Inhibition of miR-885-5p improved proliferation of non-metastatic HCC cells. Furthermore, we disclosed that miR-885-5p targeted gene encoding β-catenin *CTNNB1*, leading to decreased activity of the Wnt/β-catenin signaling pathway. The present study indicates that miR-885-5p suppresses the metastasis of HCC and inhibits Wnt/β-catenin signaling pathway by its *CTNNB1* target, which suggests that miR-885-5p to be a promising negative regulator of HCC progression and as a novel therapeutic agent to treat HCC.

## INTRODUCTION

Hepatocellular carcinoma (HCC) is the fifth most common cancer and the third most frequent cause of death from cancer globally [1]. HCC is one of the most fatal cancers in Asia and Africa. In western countries, the incidence of HCC is lower, although it is currently on the increase. The pathogenesis of HCC is a multistage process that is usually related to chronic inflammation, which is often caused by hepatitis B or hepatitis C virus infection and cirrhosis [2, 3]. Late diagnosis, metastasis and chemoresistance all hinder the effective treatment of HCC. Therefore, an urgent need exists to elucidate the molecular and biological basis of HCC and to identify early diagnostic classifiers that can reliably stratify patients for therapy.

MicroRNAs (miRNAs) are ~22 nucleotide long, endogenous, noncoding RNA molecules that suppress gene expression by binding to their target mRNAs. MiRNAs are involved in many different biological processes, including cell proliferation, differentiation, and apoptosis. These key biological functions suggest that miRNAs might contribute to regulation of the pathological processes of many diseases, including cancer. In this regard, some studies have reported that the miRNA signature was used for tumor grading differentiation and diagnosis biomarkers [4–9]. Previous studies suggested that some miRNAs function as tumor suppressors, such as let-7 [10], miR-101 [11], miR-122 [12], miR-29b [13], miR-195 [14] and miR-125b [15]. However, other miRNAs act as oncogenes, such as miR-17-5p [16], miR-18a [17], miR-106a[18] and miR-221/222 [19]. Some miRNAs are even associated with the clinicopathological features of HCC, such as hepatitis B and C virus infection [20, 21], cirrhosis [22], tumor growth [23], metastasis [24] and prognosis [25, 26]. In fact, miR-885-5p was first identified from a pheochromocytoma in 2010 [27], and we previously reported that circulating miR-885-5p is present at higher levels in patients with cirrhosis or HCC than in healthy, control individuals [28]. Subsequently, Yan et al. reported that miR-885-5p reduced MMP-9 expression, which mediates the invasion of glioblastomas [29]. miR-885-5p was found to be downregulated in neuroblastomas and to bind 3′UTR of CDK2 and MCM5 [30]. Recently, miR-885-5p was reported as a diagnosis and prognosis factor in plasma or whole blood in detection of pancreatic cancer [31, 32]. Although the function of miR-885-5p has been studied *in vitro* in a few cancer cell lines; however, the functions and mechanisms of miR-885-5p in the progression of HCC remain yet unclear.

In this study, we investigated the relationship between levels of miR-885-5p in HCC patient specimens or cell lines and the TNM stage or metastatic potential of this cancer. In addition, we determined that miR-885-5p suppressed metastasis of HCC *in vitro and in vivo*. We explored the possible molecular mechanism underlying the functions of miR-885-5p in HCC is that *CTNNB1* is a novel target gene of miR-885-5p. We showed that ectopic miR-885-5p downregulated the activity of Wnt/β-catenin signaling pathway. Taken together, these data are likely to indicate that miR-885-5p is a potential biomarker for the diagnosis of HCC as well as a therapy target for the development of novel therapies to treat HCC.

## RESULTS

### Downregulated expression of miR-885-5p in highly malignant HCC tissues and cell Lines

**Figure 1:**
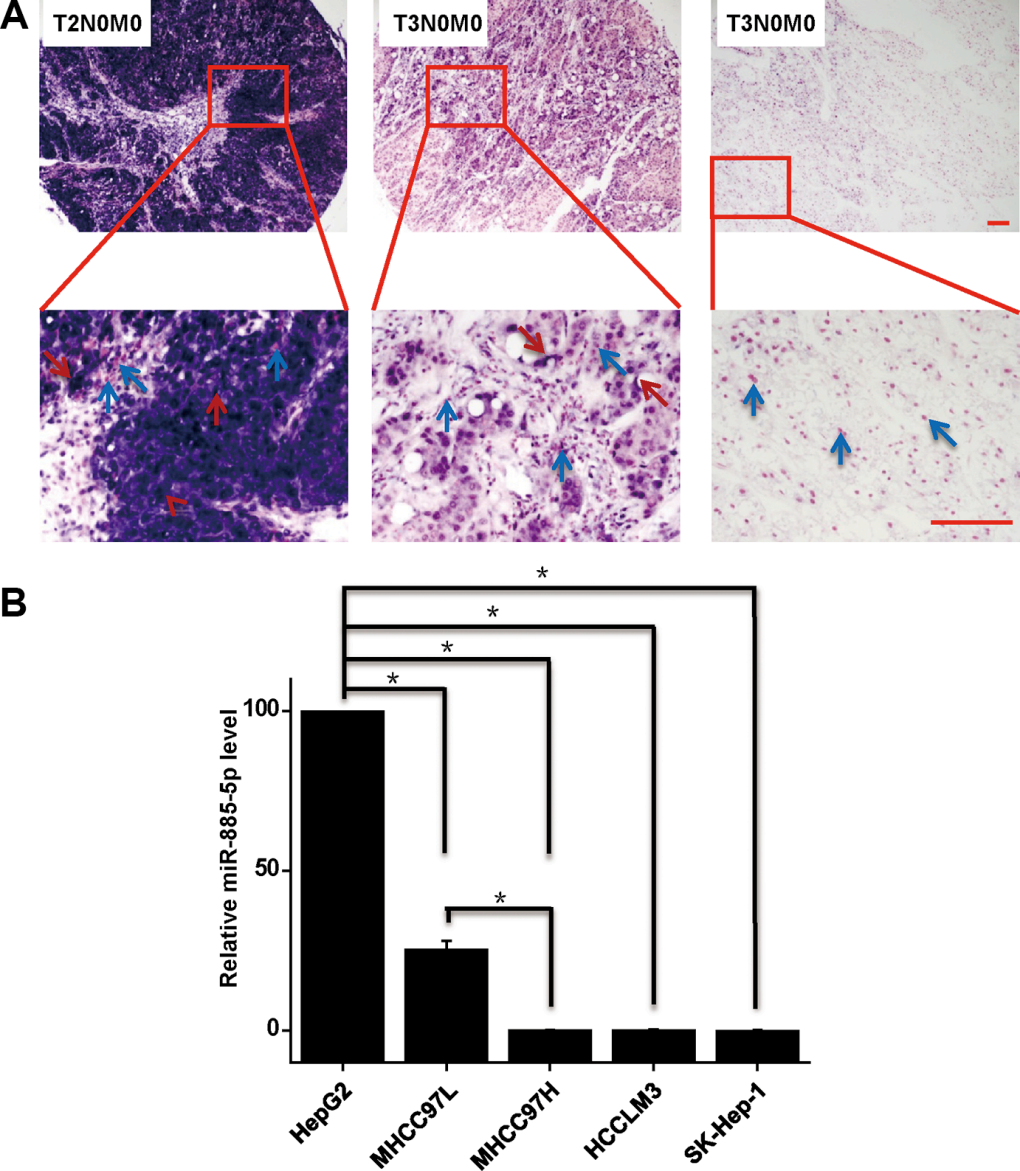
Expression of miR-885-5p is downregulated in highly malignant HCC tissues and cell lines (**A**) *In situ* hybridization of miR-885-5p in 227 cores HCC tissue array, including 81 T2N0M0 staged HCC tissues and 146 T3N0M0 staged HCC tissues. Cytoplasmic miR-885-5p are denoted by red arrowheads, and nucleus are denoted by blue arrowheads. (**B**) Real-time qRT-PCR of miR-885-5p was performed with a nonmetastatic HCC cell line HepG2 and several metastatic cell lines. Scale bars = 100 μm. ^*^P < 0.05.

Expression of miR-885-5p in HCC was performed by *in situ* hybridization tissue arrays that contained 227 cores of HCC tissue from 227 patients. Results were classified as either negative, moderate (0% to < 25%) or strong (> 25%). Most HCC tissues expressed different levels of miR-885-5p (Figure 1A). To determine whether miR-885-5p is differentially expressed in the HCC tissues of different TNM stages of tumor development, the relationship between expression of miR-885-5p and clinicopathology was analyzed by either the *χ*^2^ method or Fisher's exact test. Patients in stage II (*n* = 81) exhibited higher expression of miR-885-5p than those in stage III (*n* = 146; *P* < 0.001). There was no significant relationship between miR-885-5p expression and other clinicopathological factors, such as histological grade, gender and age (*P* > 0.05; Table 1).

**Table 1:** Correlation between miR-885-5p expression and pathological grading of HCC

	Total	Negative	Moderate	Strong	*P* value
Age					0.452
<50	140	6	44	90	
≥ 50	86	7	24	56	
Gender					0.494
Male	178	11	56	111	
Female	49	2	12	35	
Histological grade					0.074
1	20	2	11	7	
2	149	8	40	101	
3	58	3	16	39	
Tumor stage					< 0.001
II	81	2	12	67	
III	146	11	58	77	

Next, we evaluated levels of miR-885-5p in various HCC cell lines by using Real-time qRT-PCR. Levels of miR-885-5p were decreased in higher metastatic HCC cell lines (Figure 1B). These data suggest that miR-885-5p levels were downregulated during the progression of HCC metastasis.

### Expression of miR-885-5p correlates with survival of HCC patients

**Figure 2:**
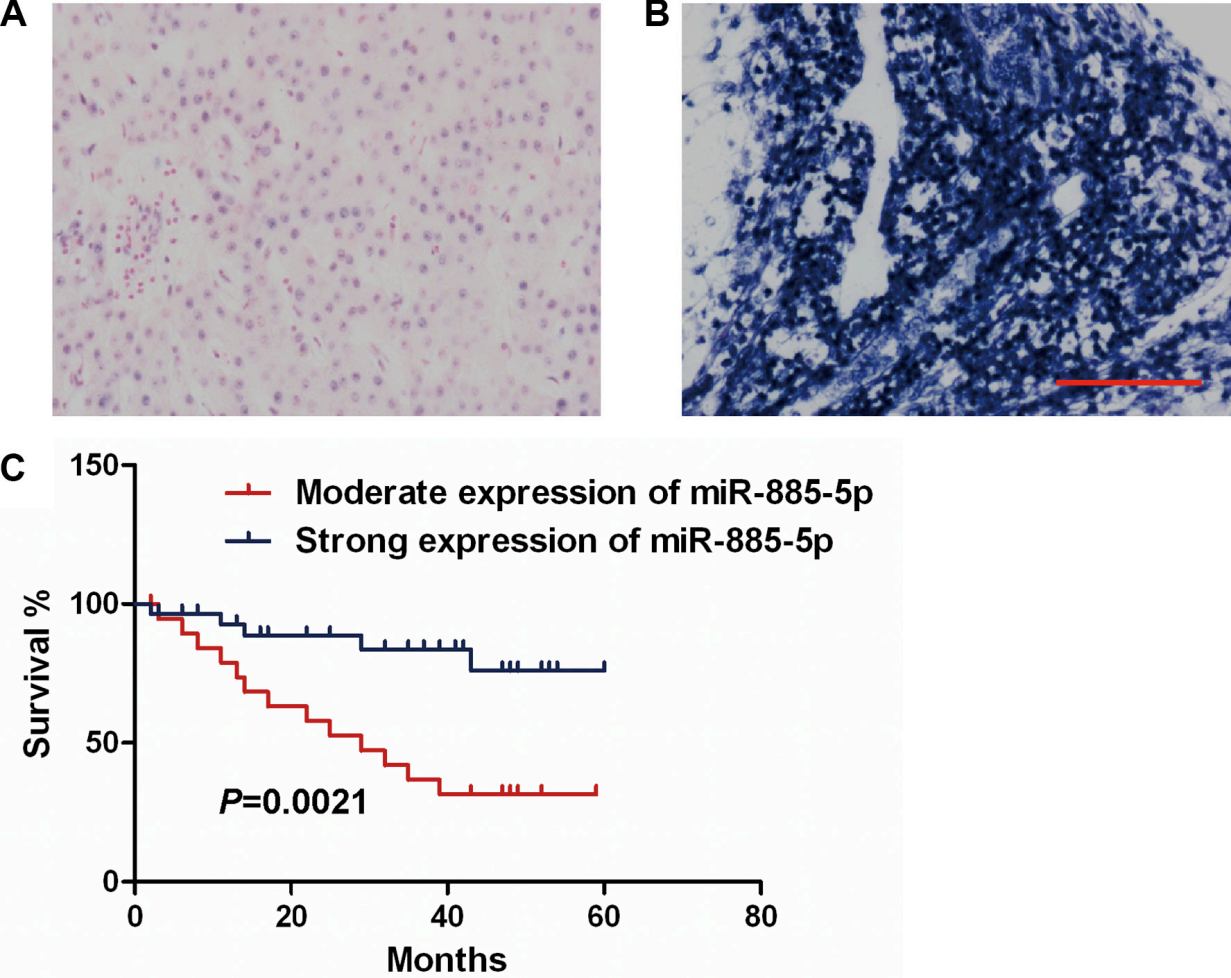
Low miR-885-5p expression correlates with poor survival of HCC patients (**A**) Moderate expression of miR-885-5p in HCC tissues. (Scale bar = 100 μm). (**B**) High expression levels of miR-885-5p in HCC tissues. (**C**) Kaplan-Meier polts illustrate that survival is based on the expression of miR-885-5p.

In an effort to further investigate whether the deregulated expression of miR-885-5p correlates with the survival of HCC patients, the expression levels of miR-885-5p were determined in 51 HCC samples from 51 individual HCC patients (Figure 2A and 2B). Kaplan-Meier survival analyses showed that the higher miR-885-5p levels in the HCC tissues significantly correlated with the markedly prolonged overall survival of these HCC patients (Figure 2C). The findings suggest that miR-885-5p may be a biomarker for HCC prognosis.

### miR-885-5p inhibits HCC metastasis and growth *in vitro* and *in vivo*

**Figure 3:**
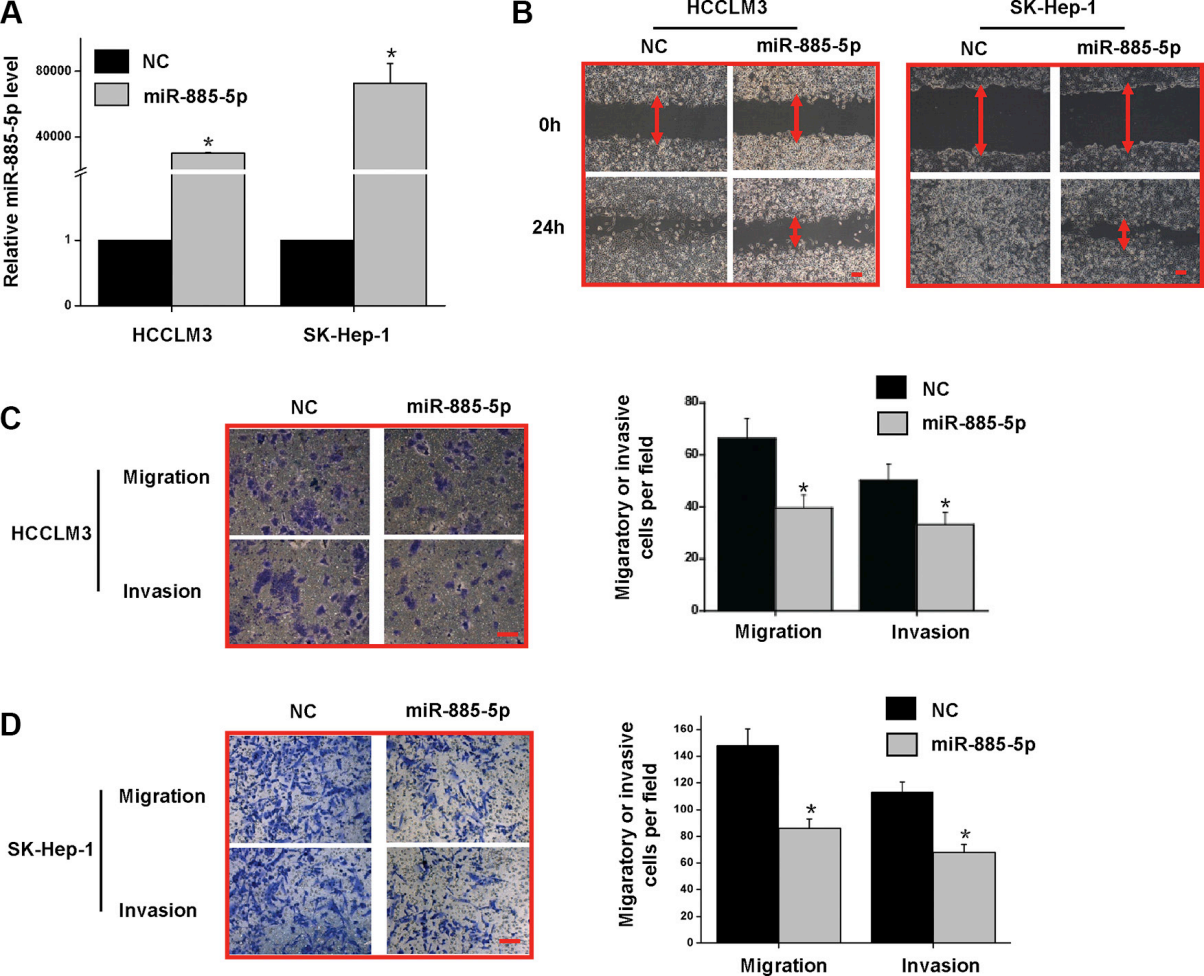
Overexpression of miR-885-5p in HCCLM3 and SK-Hep-1 reduced the migratory and invasive potential *in vitro* (**A**) miR-885-5p expression levels after treatment of HCC cell lines with either miR-885-5p mimics or miR-NC (negative control). (**B**) Wound healing assays in the HCCLM3 and SK-Hep-1 cells expressing either miR-885-5p or miR-NC. (**C)** and (**D**) Transwell migration and invasion assays in HCCLM3 (C) and SK-Hep-1 (D) cells expressing miR-885-5p or miR-NC. The representative images are shown on the left, and the quantification of five randomly selected fields on the right. These values shown are expressed as the mean ± SD. The scale bar = 100 μm. ^*^P < 0.05.

To better understand the biological functions of miR-885-5p in the progression of HCC, we overexpressed miR-885-5p in HCC cell lines, HCCLM3 and SK-Hep-1, which have high metastatic and malignant properties. Levels of miR-885-5p were 1,000-fold higher in cells after transfection with the miR-885-5p mimics than those transfected with the control miRNA (Figure 3A). Wound healing assay demonstrated that miR-885-5p suppressed cell migration in HCCLM3 and SK-Hep-1 cells (Figure 3B). Furthermore, we examined the role of miR-885-5p in the mobility and invasiveness of HCC cells using transwell chambers with or without Matrigel. The transwell assays without Matrigel clearly indicated that miR-885-5p clearly suppresses migration of HCCLM3 and SK-Hep-1 cells when compared to control groups (Figure 3C and 3D). The invasiveness of miR-885-5p-expressing HCCLM3 and SK-Hep-1 cells was downregulated as demonstrated by transwell assays with Matrigel. Together, these results indicate that miR-885-5p significantly inhibited HCC cell migration and invasion *in vitro*.

Since it was previously reported that miR-885-5p can inhibit proliferation of neuroblastoma [30], we used HepG2 cells to assess whether miR-885-5p could also decrease the proliferation of these cells (Supplementary Figure S1A and S1B). In fact, we observed that upon miR-885-5p down-regulation, proliferation of HepG2 cells increased, suggesting that miR-885-5p also suppressed HCC growth *in vitro*.

**Figure 4:**
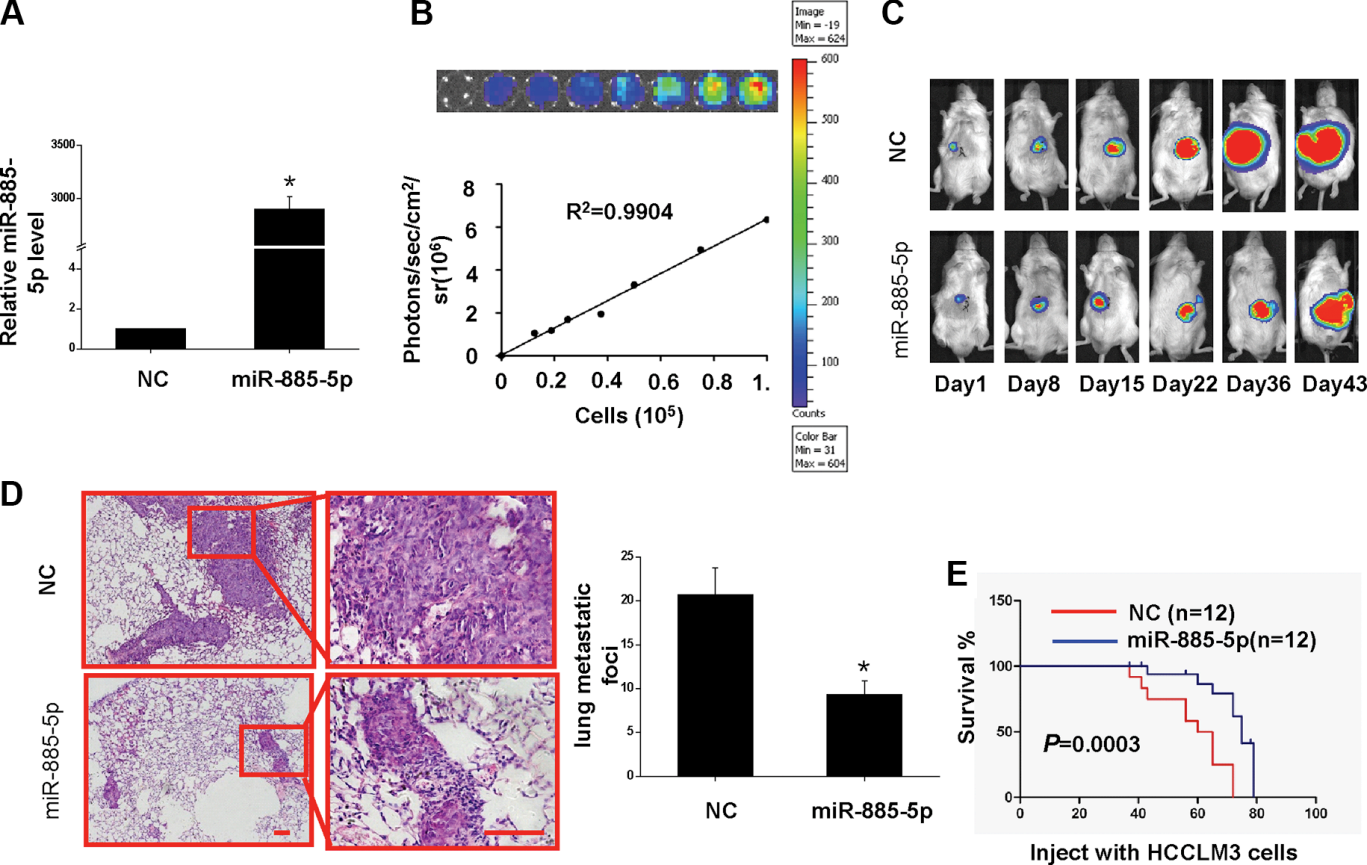
Overexpression of miR-885-5p inhibits proliferation of HCCLM3 cells *in vivo* and prolongs the survival of tumor-bearing Mice (**A**–**D**) HCCLM3-lv- miR-885-5p or HCCLM3-lv-NC tumor-bearing mice were euthanized after 43 days, and the lungs were histologically examined for metastatic foci. (A), miR-885-5p expression levels of HCCLM3-lv-miR-885-5p and HCCLM3-lv-NC cells. (B), *Ex vivo* imaging analysis of transfected HCCLM3 cells indicates increasing bioluminescence signals with increasing cell number (R^2^ = 0.9904). (C), *In vivo* imaging of tumors over 43 days. (D), Number (mean ± SD) of lung metastatic foci was determined from five serial sections. Values shown are expressed as mean ± SD. The scale bars = 100 μm. *P < 0.05. (**E)**, Survival curve for mice injected with HCCLM3 cells expressing miR-885-5p or NC control (negative control) as determined by Kaplan-Meier analysis.

In an effort to investigate the anti-tumor effects of miR-885-5p *in vivo*, Luciferase-labeled HCCLM3/lv-miR-885-5p cells were followed for 43 days. Firstly, two Luciferase-labeled stable cell lines were established, HCCLM3/lv-miR-885-5p and HCCLM3/lv-NC (Figure 4A and 4B). Next, orthotopic liver implantation of HCCLM3/lv-miR-885-5p and HCCLM3/lv-NC cells into NOD/SCID mice was performed. Bioluminescence signals were monitored one week after implantation of HCC cells, followed by increasing signals as the tumor progressed to day 43. The result showed miR-885-5p restoration significantly repressed the growth of the HCCLM3 cells (Figure 4C). Moreover, both number and size of metastatic nodules in the lung were significantly decreased in the miR-885-5p overexpression group compared to the vector group after HCCLM3 cells *in situ* were grown for 43 days (Figure 4D). Establishment of an additional set of orthotopic HCCLM3 tumors and found that miR-885-5p also prolonged the survival of the tumor-bearing mice (Figure 4E). Taken together, these results strongly suggest that miR-885-5p acted as a negative regulator of metastatic progression of HCC.

Anti-tumor effects of miR-885-5p were further investigated by subcutaneous HCC xenograft model (Supplementary Figure S2B). In fact, intratumoral injection of cholesterol-conjugated miR-885-5p mimics demonstrated that when miR-885-5p expression in SK-Hep-1 cells was elevated (Supplementary Figure S2A), whereas the tumor growth was inhibited (Supplementary Figure S2C and S2D). This result indicates that miR-885-5p inhibited not only the metastatic progression of HCC but also the growth of HCC.

### miR-885-5p downregulates β-catenin

**Figure 5:**
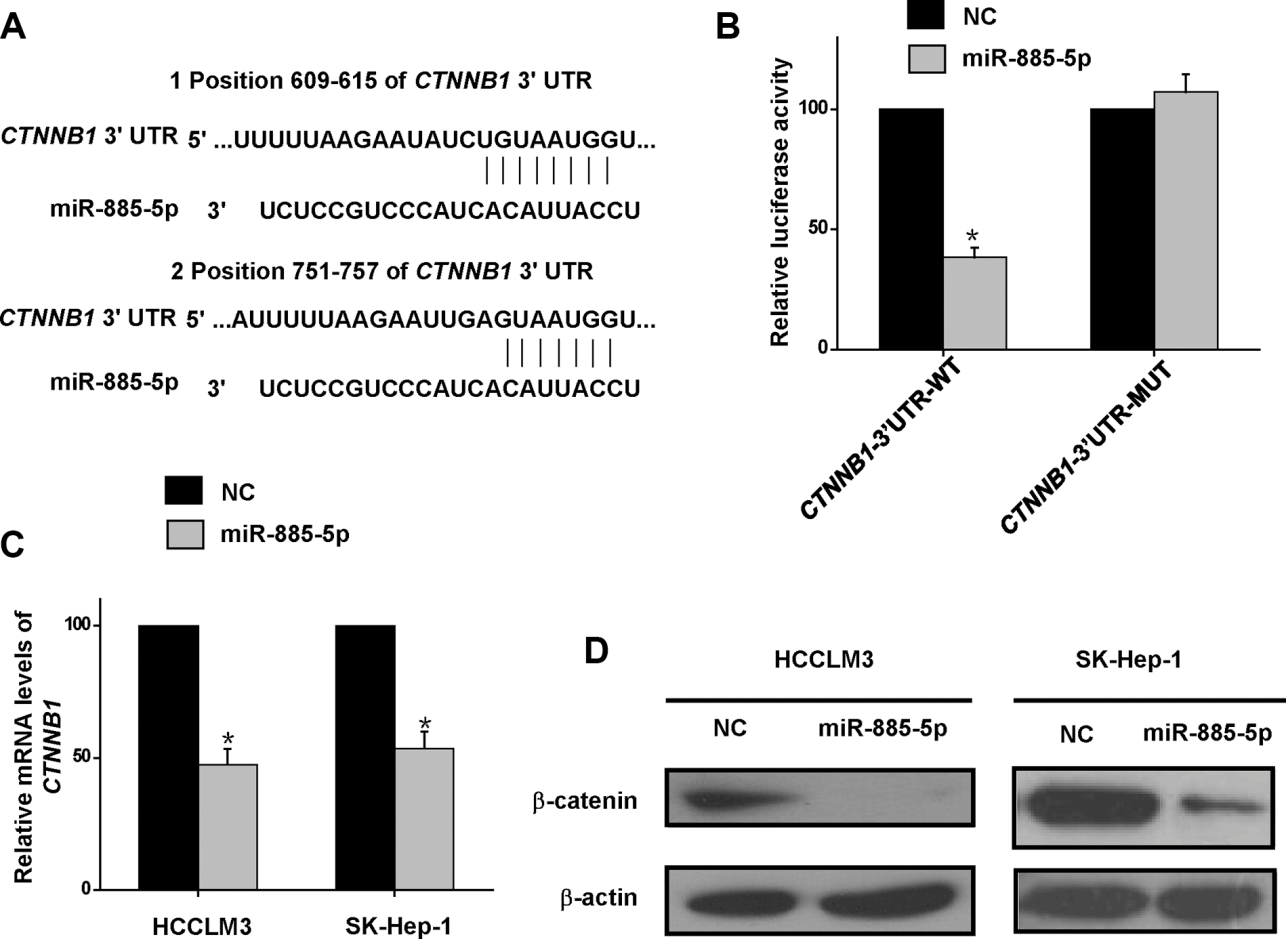
β-catenin is the direct target of miR-885-5p (**A)**, Alignment of miR-885-5p with *CTNNB1* at the 3′-UTR. (**B)**, Effect of miR-885-5p on luciferase activity of Luc-*CTNNB1*-UTR and Luc-*CTNNB1*-UTR-mut. (**C**), Real-time qRT-PCR indicating the reduced levels of *CTNNB1* when compared with the negative controls (NC) in the HCCLM3 and SK-Hep-1 HCC cell lines. (**D)**, Western blots indicated reduced levels of β-catenin in miR-885-5p mimics or miR-NC transfected HCCLM3 and SK-Hep-1 cells. Levels of β-actin are shown as an internal control. ^*^P < 0.05.

Our *in vitro* and *in vivo* results provided evidences that miR-885-5p functioned as a suppressor of HCC. An investigation was initiated to explore the molecular mechanisms responsible for the multiple functions of miR-885-5p. Since miRNAs mainly function through suppressing the expression of their target genes, potential targets were analyzed with three prediction databases, PicTar, TargetScan, and miRanda. Among the potential target genes analyzed, the β-catenin gene, *CTNNB1*, was chosen for further experimental validation (Figure 5A), mainly because it is co-transcription factor in the Wnt/β-catenin signaling pathway, which has a significant role in HCC development and progression. To verify the predicted target, we performed dual-luciferase reporter assays, which indicated that co-expression of miR-885-5p mimics in HCCLM3 cells significantly inhibited the activity of luciferase which contained the wild-type, but not the mutant, 3′-UTR of *CTNNB1* (Figure 5B). Furthermore, *CTNNB1* mRNA levels were found to be decreased by transfection of the miR-885-5p mimics into HCCLM3 or SK-Hep-1 cells (Figure 5C). Additionally, β-catenin protein levels were decreased by transfection of the miR-885-5p mimics into HCCLM3 or SK-Hep-1 cells (Figure 5D). Conversely, β-catenin protein level was upregulated by the infection of lv-miR-885-locker which caused knockdown of miR-885-5p in HepG2 cells (Supplementary Figure S3A). These data demonstrate that miR-885-5p inhibited the expression of β-catenin by directly targeting its 3′-UTR.

### miR-885-5p suppresses the Wnt/β-catenin signaling pathway

**Figure 6:**
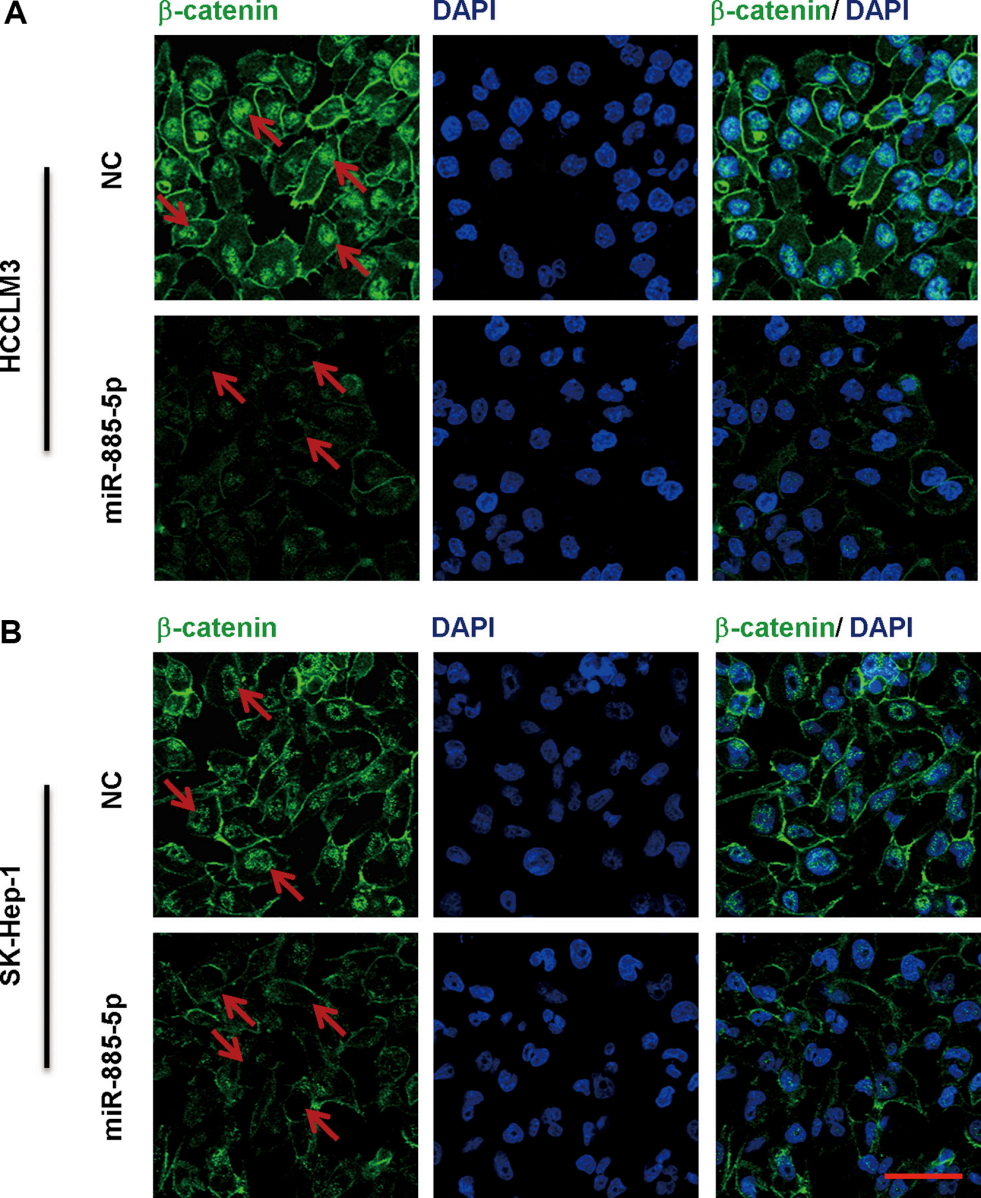
Immunofluorescence staining of β-catenin in HCC cell lines transfected with miR-885-5p Immunofluorescence staining of in HCCLM3 (**A**) or SK-Hep-1 (**B**) cells transfected with miR-885-5p mimics or NC demonstrates differential localization. The DAPI staining (blue) labels DNA in all the cells. The scale bar = 100 μm.

**Figure 7:**
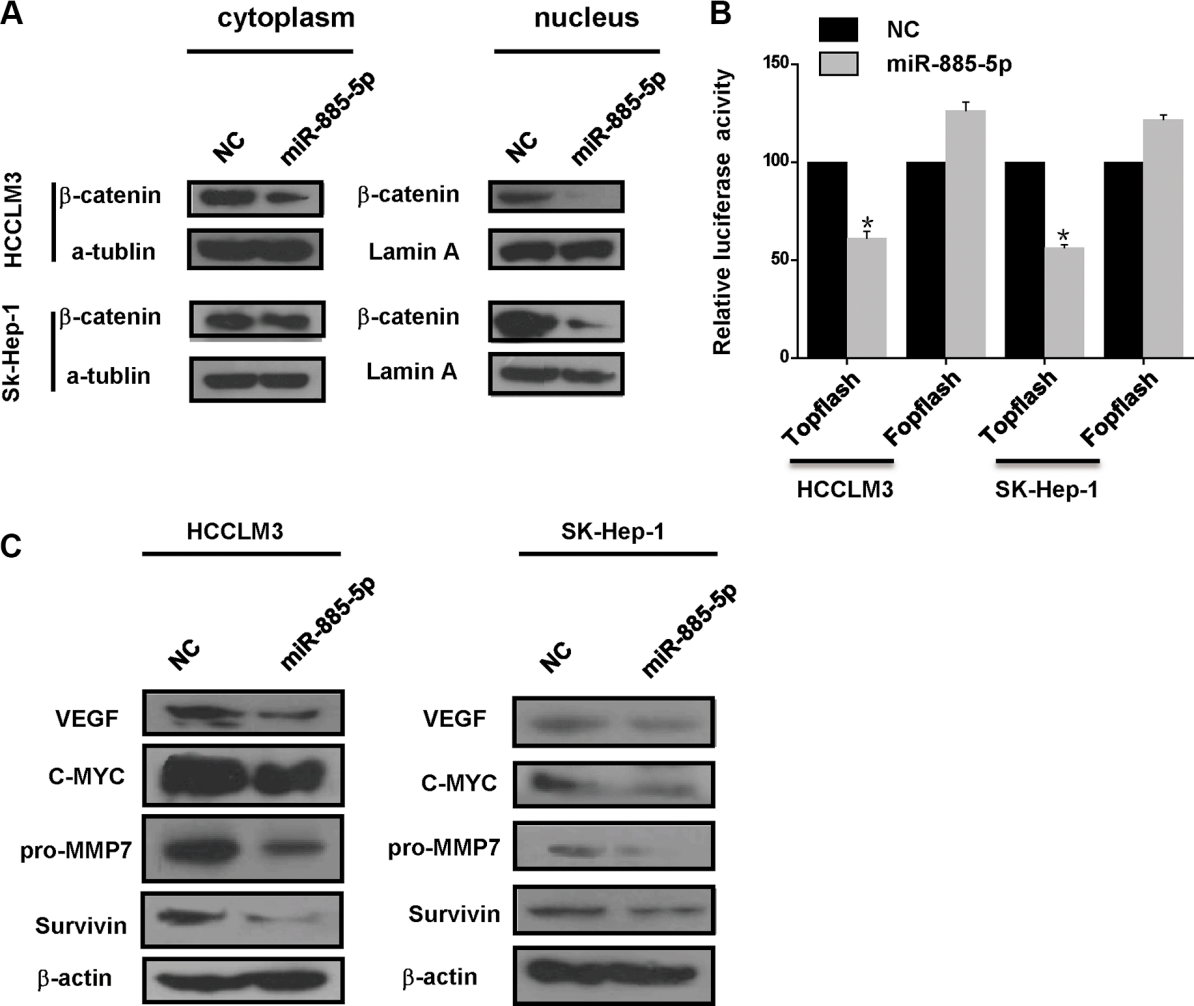
miR-885-5p regulates the Wnt/β-catenin pathway by targeting *CTNNB1* (**A)**, Western blot analysis of β-catenin in the cytoplasmic and nuclear fractions from HCCLM3 or SK-Hep-1 cells transfected with the miR-885-5p mimics or NC. Bands on the blot were normalized to α-tubulin for the cytoplasmic fraction or lamin A for the nuclear fraction. (**B**), Luciferase activity of the wild-type or mutant *CTNNB1-*3′UTR reporter gene in the HCCLM3 cells transfected with the miR-885-5p mimics or NC using the TOPflash reporter plasmid for the Wnt/β-catenin assay with FOPflash as the negative control. ^*^P < 0.05. (**C**) MYC, VEGFA, pro-MMP7 and Survivin expression in the HCCLM3 or SK-Hep-1 cells transfected with the miR-885-5p mimics or NC as measured by western blot.

To further validate that β-catenin serves as miR-885-5p binding target, it was determined whether or not miR-885-5p-mediated suppression of β-catenin expression indeed suppressed β-catenin transcriptional activity. Here, we found that control HCCLM3 cells and SK-Hep-1 cells exhibited both cytoplasmic and nuclear localization of β-catenin, whereas in the miR-885-5p-overexpressing cells, β-catenin was associated with cell-cell junctions (Figure 6). Furthermore, in cytoplasmic fraction prepared from the miR-885-5p-overexpressing HCCLM3 cells, the amount of β-catenin was decreased; therefore, a lesser amount of β-catenin accumulated in the nucleus of these cells as compared to control cells (Figure 7A). In an effort to monitor the activity of the Wnt/β-catenin signaling pathway in the cells, β-catenin reporter assays were performed using both the Topflash and Fopflash constructs. Result of these assays demonstrated that miR-885-5p decreased the β-catenin activity by ~45% (Figure 7B), whereas this activity was increased 1.5-fold (*P* < 0.05) when HepG2 cells were infected with plv-miR-885-5p-locker (Supplementary Figure S3B). In addition, we found that miR-885-5p significantly decreased the levels of the proteins, MYC, VEGF, pro-MMP7, and Survivin (Figure 7C and Supplementary Figure S3C), which are downstream proteins of Wnt/β-catenin signaling pathway. Therefore, these results confirm that miR-885-5p decreased the activity of the Wnt/β-catenin signaling pathway, possibly leading to the suppression of HCC metastasis.

### miR-885-5p expression correlated inversely with β-catenin

**Figure 8:**
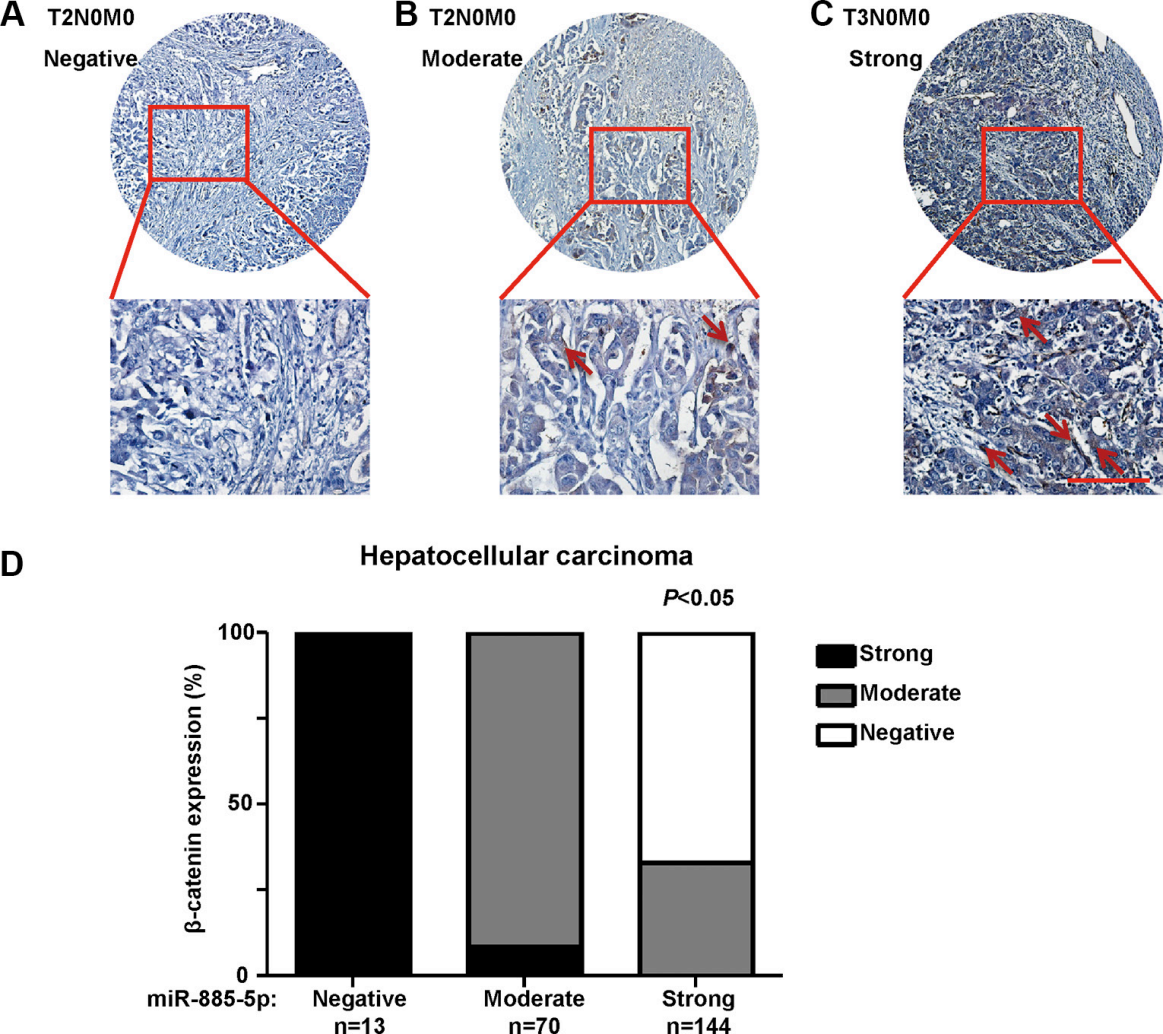
The expression of β-catenin in HCC tissues Immunohistochemistry of β-catenin expression in 227 cores HCC tissue array, including 81 T2N0M0 staged HCC tissues and 146 T3N0M0 staged HCC tissues. (**A)**, The image of T2N0M0 HCC tissue which the expression of β-catenin is negative. (**B**), The image of T2N0M0 HCC tissue which the expression of β-catenin is moderate. (**C)**, The image of T2N0M0 HCC tissue which the expression of β-catenin is strong. Staining of β-catenin are denoted by red arrowheads. (**D**) Statistical analysis of the expression relationship between β-catenin and miR-885-5p in HCC tissue array. Plots represent the percentage of tissue samples with different expression level of β-catenin (y-axis) in each group that was defined as miR-885-5p negative, weak, moderate or strong (x-axis).

To determine the relationship between miR-885-5p and expression of β-catenin in human HCC tissues, immunohistochemical analyses were conducted in the same HCC tissue microarrays as those used in miR-885-5p *in situ* hybridization with β-catenin antibody. The results showed that the protein levels of *CTNNB1* in HCC tissues with weak miR-885-5p expression were significantly higher than those in HCC tissues with strong miR-885-5p expression (Figure 8). Besides, we observed that patients in stage II (*n* = 81) exhibited lower expression of β-catenin than those in stage III (*n* = 146; *P* < 0.001; Figure 8). The results indicated that the expression level of β-catenin positively correlated with liver cancer TNM stages; however, no significant relationship between β-catenin expression and other clinicopathological factors, such as histological grade, gender and age (*P* > 0.05; Table 2).

**Table 2:** Correlation between β-catenin expression and pathological grading of HCC

β-catenin	Total	Negative	Moderate	Strong	*P*
Age					0.251
< 50	140	55	75	10	
≥ 50	87	41	37	9	
Gender					0.965
Male	178	76	87	15	
Female	49	20	25	4	
Histological grade					0.096
1	20	11	7	2	
2	149	54	80	15	
3	58	31	25	2	
Tumor stage					< 0.001
II	81	56	22	3	
III	146	40	90	16	

## DISCUSSION

Previous studies reported that miR-885-5p suppresses tumor cell proliferation and survival of neuroblastoma [30] as well as metastasis of glioblastomas [29]. Here, we demonstrate that miR-885-5p functions an anti-metastatic miRNA and a negative regulator of the Wnt/β-catenin signaling pathway, which is a key pathway in the development and progression of HCC and various other tumors. We found that the expression of miR-885-5p negatively correlates with the invasive and metastatic potential of different HCC patient's tissues and cell lines. Additionally, this phenotype led us to further study the biological effects of miR-885-5p. Based on our results, we propose that miR-885-5p inhibits metastasis of HCC.

Hou *et al*. [23] have reported that functions of miRNA abundance ratios in the entire miRNome of specific tissues may be important. They reported miR-199a/b-3p is downregulated in various HCC tissues. Notably, in our study HCC tissues and cell lines with higher metastatic properties display lower levels of miR-885-5p than less metastatic tissues and cell lines (Figure 1). These observations suggest that miR-885-5p may be a negative regulator of metastasis in HCC. To identify the protecting function, we determined whether miR-885-5p levels correlate with the survival of HCC patients. Our analyses revealed that miR-885-5p expression was a predictor of survival of HCC patients (Figure 2).

We further explored the pathophysiological functions of miR-885-5p and found that expression of miR-885-5p in the highly metastatic HCC cell lines decreased the migration and invasiveness of the cells *in vitro* (Figure 3). In this study, consistent with *in vitro* results, our animal model experiments show that upregulation of miR-885-5p also inhibits metastasis of HCC (Figure 4). Knockdown of miR-885-5p in nonmetastatic HepG2 cells indicated that lv-miR-885-5p-locker-infected HepG2 cells possess an increased ability to grow (Supplementary Figure S1). Together, such results suggest that miR-885-5p should be considered a potential target for HCC gene therapy.

To further investigate the effects of miR-885-5p in gene therapy, we used a cholesterol-conjugated, small RNA delivery system that has drug-like features. We confirmed that this technique could effectively overexpress miR-885-5p in tumors of an HCC mouse model. We found that repeated intratumor injections of miR-885-5p agomirs can actually halt HCC growth. Thus, our findings have implications for testing anti-HCC metastasis agents in clinical trials (Supplementary Figure S2).

In this study, we also found miR-885-5p in HCC inhibited the expression of β-catenin leads to suppression of the Wnt/β-catenin signaling pathway. Afanasyeva1 *et al*. reported that miR-885-5p binds *CDK2* and *MCM5*, activates p53 and inhibits proliferation and survival. Yan *et al*. showed that miR-885-5p inhibited the expression of MMP9 indirectly. In contrast, the results of our study provided strong evidence that miR-885-5p, through binding *CTNNB1*, inhibited metastasis and growth of HCC. In fact, upregulation of miR-885-5p inhibited the Wnt/β-catenin signaling pathway and expression of some of its downstream genes (Figures 5–7). Wnt/β-catenin pathway was inactive due to inhibition of β-catenin expression and translocation to nucleus (Figures 5 and 6). As we all know, in inactive Wnt/β-catenin signaling pathway, β-catenin will be translated and subsequently phosphorylated by destruction complex, which contains axin, glycogen synthase kinase 3β (GSK3β), adenomatous polyposis coli (APC) and casein kinase 1 (CK1). Phosphorylation of Ser and Thr residues in β-catenin will trigger proteasomal degradation of β-catenin [33]. miR-885-5p may suppress the form of destruction complex indirectly, and this suppression results in absence of β-catenin in nuleus; however, this mechanism will be further studied. Conversely, downregulation of miR-885-5p increased the activity of its Wnt/β-catenin signaling pathway and expression of its downstream genes (Supplementary Figure S3). To further study the correlation between the expression of β-catenin and clinicopathology, we analyzed the HCC tissue array by immunohistochemistry, and the tissues were the same as those used in Figure 1. Statistical analysis showed that the expression of β-catenin strongly correlated with TNM stages. The expression trend of β-catenin was conversely with miR-885-5p (Figure 8) (Table 2). We also analyzed the correlation between β-catenin status and the survival of patients. The results showed that the expression levels of β-catenin inversely correlated with the patients' survival lengths (Supplementary Figure S4). Our study shows that the lower miR-885-5p levels or higher β-catenin levels in the HCC tissues significantly correlated with poorer overall survival of the HCC patients.

In summary, our findings significantly suggest a novel mechanism for downregulation of the Wnt/β-catenin pathway activity. This has critical imllications on tumor progression in HCC. Our study provides *in vitro* and *in vivo* evidence to support a rationale for developing miR-885-5p as a therapeutic agent to treat HCC-related metastasis.

## MATERIALS AND METHODS

### Tissue microarrays and cell lines

The liver tissue microarrays for determining miR-885-5p expression were purchased from Chaoying Biotechnology (Xi'an, China). The liver tissue microarray for survival analysis was purchased from Xinchao Biotechnology (Shanghai, China). The human HCC cell lines, HepG2 and Sk-Hep-1, were obtained from ATCC, and the metastatic human HCC cell lines, MHCC97L, MHCC97H and HCCLM3, were obtained from the Liver Cancer Institute of Fudan University. These cell lines were cultured in Dulbecco's modified Eagle's medium (DMEM; Invitrogen, Carlsbad, CA) with 10% fetal bovine serum (FBS) and antibiotics at 37°C in a 5% CO_2_ atmosphere.

### Vector constructs

The miR-885-5p lentiviral expression vector, the miR-885-5p-locker vector and the packaging vectors, plv-PACK-1, plv-PACK-2 and plv-PACK-1, were purchased from Biostettia Biotechnology (San Diego, CA). The firefly luciferase reporter plasmids contained the wild-type and mutant 3′-UTR segments of human *CTNNB1* genes as follows: psiCHECK2-*CTNNB1*-3′UTR-WT and psiCHECK2-*CTNNB1*-3′UTR-MUT. Briefly, *CTNNB1* 3′UTR containing the response element of miR-885-5p was amplified by PCR with the cloning primers *CTNNB1*-WT-F: 5′-CCGCTCGAGCTGACACACTAACCAAGCTG-3′ and *CTNNB1*-WT-R: 5′-ATTTGCGGCCGCTCCACCTGCTTA TTTTAAGC-3′. Additionally, the mutant3′-UTR of *CTNNB1* with site-specific mutations in the response element of miR-885-5p was created by site-directed mutagenesis using the primers *CTNNB1*-MUT-F: 5′-TTTTTTTTAAGAATATCTGATTACGTACTGACTTT CTTGCTTT-3′ and CTNNB1-*MUT*-R: 5′-AAAGCAAGAA AGTCAGTACGTAATCAGATATTCTTAAAAAAAA-3′. The 3′-UTRs (3′-UTR WT and 3′-UTR MUT) were cloned into the psiCHECK2 vector (Promega,Madison, WI, USA) using XhoI and NotI restriction enzymes.

### Lentiviral-mediated overexpression or inhibition of miR-885-5p

Lentivirus was produced as previously described [34]. Lentivirus was produced by co-transfecting 293T cells according to the manufacturer's instructions (Biostettia, San Diego, CA). The vectors were described in the Vector Constructs section. The HCC cells were infected with the lentiviral supernatant for 1 h in the presence of 8 μg/ml polybrene. Two days after infection, the selection antibiotic, puromycin (4 μg/ml) or blasticidin (4 μg/ml), was added to the culture medium, and the cell populations selected after 2 weeks.

### Reagents

The antibodies specific for MYC and VEGF were purchased from Santa Cruz Biotechnology (Santa Cruz, CA,). The antibodies specific for β-catenin was purchased from Cell Signaling Technology (Danvers, MA). The antibodies specific to Survivin and MMP7 were obtained from Abcam (Cambridge, UK). The miR-885-5p mimics and the negative controls were purchased from Applied Biosystems (Carlsbad, CA). The cholesterol-conjugated miR-885-5p mimics and the respective negative controls for *in vivo* RNA delivery were obtained from Ribobio Co. (Guangzhou, China)

### RNA extraction and real-time quantitative reverse-transcription PCR

Total RNA was extracted with the mirVana PARIS kit (Ambion, Foster City, CA) according to the manufacturer's instructions. TaqMan miRNA assays (Applied Biosystems, Foster City, CA) were used to determine the miR-885-5p levels using quantitative real-time PCR (qRT-PCR). Reverse-transcribed complementary DNA was synthesized with 5 ng total RNA using TaqMan MicroRNA Reverse Transcription Kit (Applied Biosystems, Foster City, CA). miRNA expression was normalized to U6 RNA expression.

### Oligonucleotide transfection

The miR-885-5p mimics were purchased from Ambion (Foster City, CA). The cells were seeded onto six-well culture plates and transfected the next day, cells were transfected with 50 nM miR-885-5p mimics or negative control microRNAs (miR-NC). After 72 h, cells were harvested for transwell migration assays, invasion assays or luciferase reporter assays.

### *In situ* hybridization

*In situ* detection of miR-885-5p in the patient HCC and healthy control samples was performed on a paraffin-embedded tissue microarray using a miRCURY LNA™ microRNA *in situ* hybridization (ISH) Optimization Kit for formalin-fixed, paraffin-embedded samples (Exiqon) according to the manufacturer's instructions.

### Immunohistochemistry

Expressions of β-catenin in the liver tissue microarrays were detected by standard biotin–avidin complex method with antibody against β-catenin at a 1:200 dilution. Images were recorded by Olympus BX51 Epifluorescent microscopy under a ×40 objective (Olympus Co. Tokyo, Japan). Expression levels of β-catenin in the tissue microarrays were scored according to the percentage of β-catenin positive cells in each liver tissue. Specifically, negative, 1–49% was moderate, and ≥ 50% was judged as strong immunostaining intensity.

### Western blot

Western blots were performed as previously described [35]. Briefly, the total cell lysates were prepared by lysis of cells with the radioimmunoprecipitation assay (RIPA) buffer, and protein concentrations were determined with the bicinchoninic acid (BCA) protein assay reagent (Pierce, IL, USA). After determination ‘protein concentration, 30 μg of each sample was separated on 10% SDS polyacrylamide gels and transferred to polyvinylidene fluoride (PVDF) membranes. After blocking with 5% skimmed milk for 1 h, membranes were incubated with an appropriate dilution of the primary antibody. The secondary antibodies coupled to horseradish peroxidase were visualized by enhanced chemiluminescence (Millipore, Billerica, MA). All western blots were repeated at least three times.

### Luciferase reporter assay

293T cells were seeded onto 24-well plates and transfected 12 h later with the Lipofectamine 2000 reagent (Invitrogen, Carlsbad, CA) according to the manufacturer's instructions. The miR-885-5p mimics (50 nM/well; Applied Biosystems, CA) were co-transfected with 100 ng/well luciferase reporter plasmids and 2 ng/well plasmid pRL-TK. Plasmid pRL-TK served as an internal control for the relative luciferase activity using a dual-luciferase assay reporter system (Promega, WI). For the TOPflash reporter gene assay, the cells were transfected with pRL-TK, TOPflash (wild type; Upstate, Lake Placid, NY) or FOPflash (mutant; Upstate, Lake Placid, NY). The luciferase activity in the transfected cells was measured by Dual-Luciferase Reporter Assay Kit (Promega, WI) according to the manufacturer's instructions, and the firefly luciferase activity was normalized to the renilla luciferase activity.

### *In vitro* migration and invasion assay

We performed transwell assays as our previous study [36]. In the transwell migration assay, 5 × 10^4^ cells were placed in the top chamber of each insert (Millipore, Billerica, MA) with an uncoated membrane. For the invasion assay, 8 × 10^4^ cells were placed in the upper chamber of each insert coated with 100 μl Matrigel (BD Biosciences, MA) to form a matrix barrier. For both assays, cells were trypsinized and resuspended in 200 μl DMEM, and 500 μl DMEM supplemented with 10% FBS was added to the lower chamber. After incubation at 37°C, any cells remaining in the top chamber or on the upper membrane of the inserts were carefully removed. For the migration assays, the SK-Hep-1 cells were incubated for 24 h and the HCCLM3 incubated for 48 h. For the invasion assays, SK-Hep-1 cells incubated for 36 h and the HCCLM3 were incubated for 72 h. After fixation and staining in a dye solution containing 0.1% crystal violet, the cells adhering to the lower membrane of the inserts were counted and imaged with an IX71 inverted microscope (Olympus Corp., Tokyo, Japan).

### Tumor xenograft animal model assays

Two independent sets of tumor xenograft experiments were performed.

For the *in vivo* metastasis assay, an orthotopic liver xenograft model was used. Either HCCLM3/lv-miR-885-5p or HCCLM3/lv-NC cells (5 × 10^6^) were injected subcutaneously (S.C.) into the upper left flank region of 12 NOD/SCID mice. When the S.C. tumors had grown to ~1 cm in diameter, mice were euthanized, and tumor tissues removed and implanted into the livers of two groups of NOD/SCID mice (six mice per group). These animals were euthanized after 43 days, and their tumors or livers dissected, fixed in formalin, embedded, sectioned serially, stained with hematoxylin and eosin, and viewed under a microscope. Whenever metastatic HCC cells were found on any slide of the lung sections from a mouse, this particular that animal was considered positive for lung metastases.

For the survival assay, an orthotopic liver xenograft model was applied. We performed the same procedures described above with 12 mice per group.

### Statistical analysis

Unless otherwise noted, the data are presented as the mean ± the standard deviation (SD). The differences between the groups were analyzed using a Student's *t*-test when only two groups were compared. The significance was determined by a x^2^ test as shown in Table 1 and Table 2. The correlation between miR-885-5p expression and patient survival was determined by the Kaplan–Meier method. All statistical tests were two-sided and analyses were performed with the SPSS software 17.0 (SPSS Inc., Chicago, IL).

## SUPPLEMENTARY MATERIALS FIGURES


