# Supplementary material for: miR-885-5p suppresses hepatocellular carcinoma metastasis and inhibits Wnt/β-catenin signaling pathway
Source: Oncotarget. 2016 Oct 12;7(46):75038–51. doi: 10.18632/oncotarget.12602 (PMC5342721; doi:10.18632/oncotarget.12602)
Supplement: Supplementary file 1 [file oncotarget-07-75038-s001.pdf]

miR-885-5p suppresses hepatocellular carcinoma metastasis and inhibits Wnt/β-catenin signaling pathway

Supplementary Materials

Analysis of proliferation with the cell counting kit -8 (CCK-8)

Impact of silencing miR-885-5p on HepG2 cell proliferation was measured with the Cell Counting Kit-8 (CCK-8; Dojindo Molecular Technologies, Kumamoto, Japan) according to the manufacturer's instructions. Briefly, 5000 cells/well were seeded onto 96-well plates. The following day, 10 µl CCK-8 reagent was added to each well. After 1 hour incubation at 37°C, the absorbance was measured at 450 nm. All experiments were performed in quintuplicate and each experiment was repeated at least three times.

Animal model

For the *in vivo* metastasis assay, an orthotopic liver xenograft model was used. Either HCCLM3/lv-miR-885-5p or HCCLM3/lv-NC cells (5×10^6) were injected subcutaneously (S.C.) into the upper left flank region of 12 NOD/SCID mice. When the S.C. tumors had grown to ~1 cm in diameter, mice were euthanized, tumor tissues removed and implanted into the livers of two groups of NOD/SCID mice (six mice per group). These animals were euthanized after 43 days, and their tumors or livers dissected, fixed in formalin, embedded, sectioned serially, stained with hematoxylin and eosin, and viewed under a microscope. Whenever metastatic HCC cells were found on any slide of the lung sections from a mouse, this particular that animal was considered positive for lung metastases.

For the subcutaneous injection of miR-885-5p RNA into the xenografts, SK-Hep-1 cells (5×10^6) were resuspended in 200 µl PBS and then injected subcutaneously into either side of the left flank of 6 week old NOD/SCID mice. Ten days after the inoculation, the subcutaneous SK-Hep-1 tumors were injected intratumorally with either cholesterol-conjugated miR-NC or miR-885-5p RNA, a total of six times over three weeks, with 2 nM RNA in 100 µl PBS. The tumor growth was examined over 30 days.

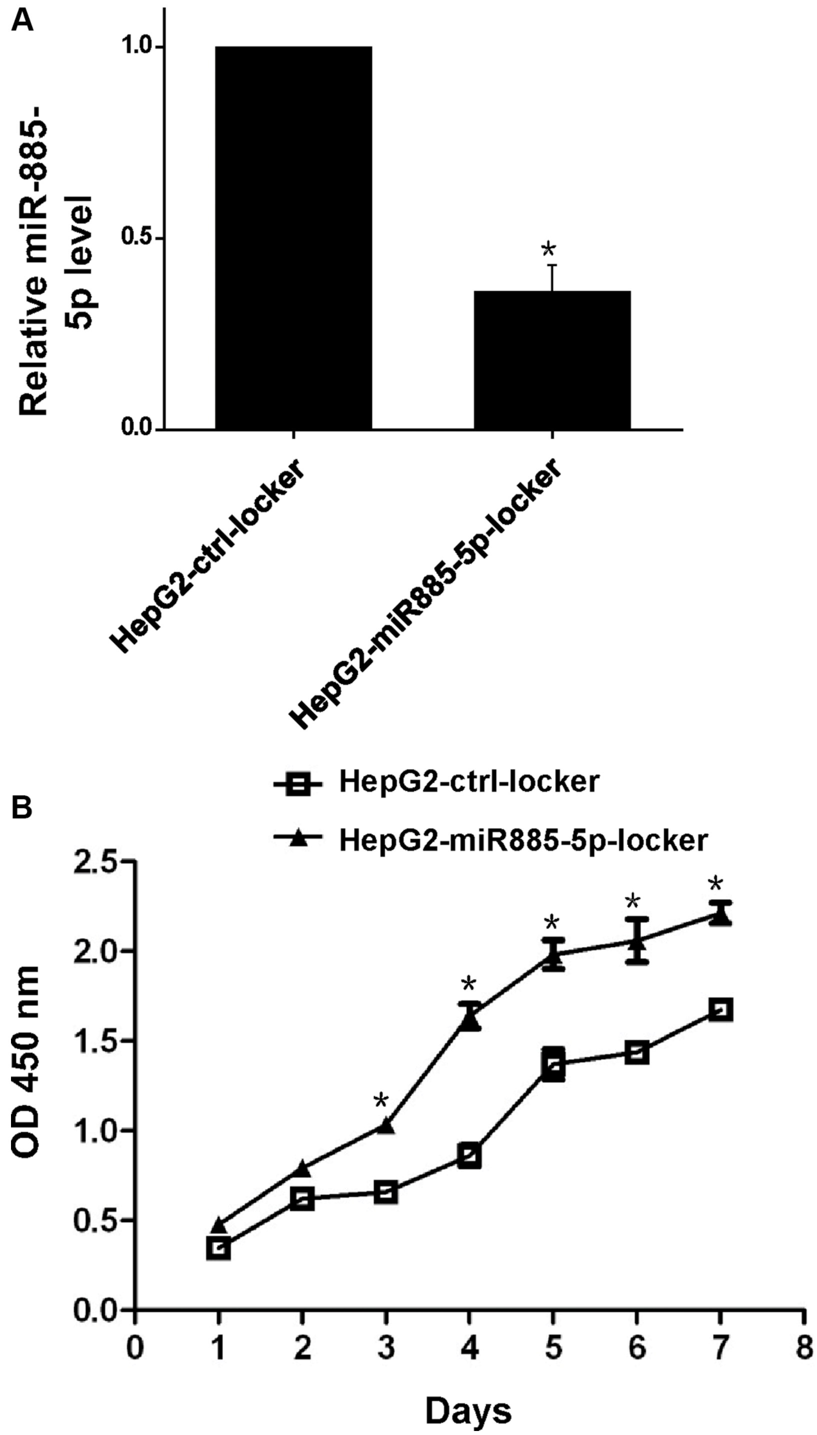

Supplementary Figure S1: Knockdown of miR-885-5p promotes HepG2 cell growth. (**A**) miR-885-5p expression levels of HepG2 cells infected with lv-miR-885-5p-locker or lv-ctrl-locker (scramble control). $^{*}P < 0.05$. (**B**) CCK-8 assays in HepG2 cells infected with lv-miR-885-5p-locker or lv-ctrl-locker.

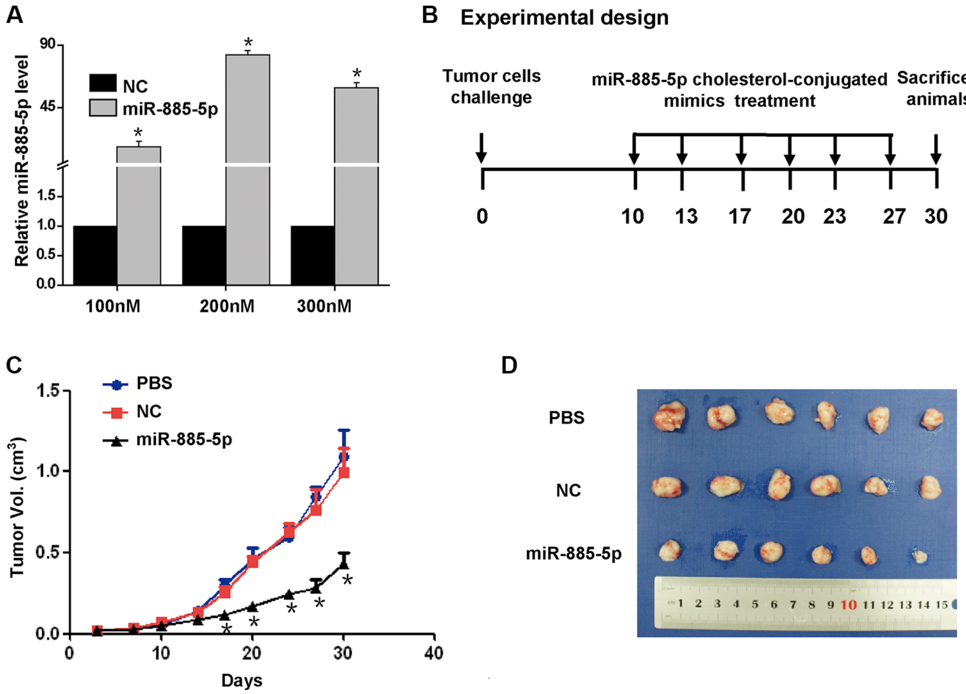

Supplementary Figure S2: Transfection with miR-885-5p cholesterol-conjugated mimics inhibits HCC growth *in vivo*. (**A**) Real-time qRT-PCR analysis of miR-885-5p expression in SK-Hep-1 cells 72 h after transfection with either the cholesterol-conjugated miR-885-5p cholesterol-conjugated mimics or control cholesterol-conjugated mimics. (**B**) A schematic of this experiment. (**C**) The effect of miR-885-5p restoration on SK-Hep-1 tumor growth. (**D**) The tumors from the three groups are shown. *$P < 0.05$.

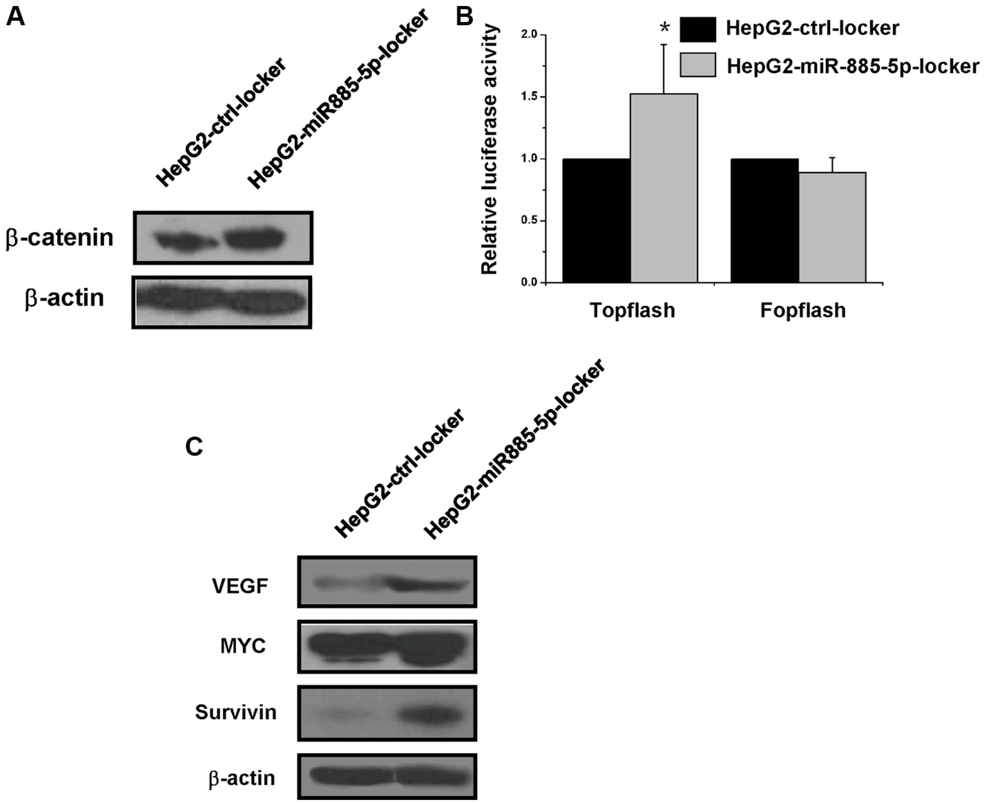

Supplementary Figure S3: Knockdown of miR-885-5p up-regulates the Wnt/β-catenin signaling pathway in HepG2 cells. (**A**) β-catenin expression in HepG2 cells infected with lv-miR-885-5p-locker or lv-ctrl-locker were measured by western blot. (**B**) Wnt/β-catenin signaling pathway activity assays in HepG2 cells infected with lv-miR-885-5p-locker or lv-ctrl-locker. *$P < 0.05$. (**C**) MYC and VEGFA expression in HepG2 cells infected with lv-miR-885-5p-locker or lv-ctrl-locker were measured by western blotting.

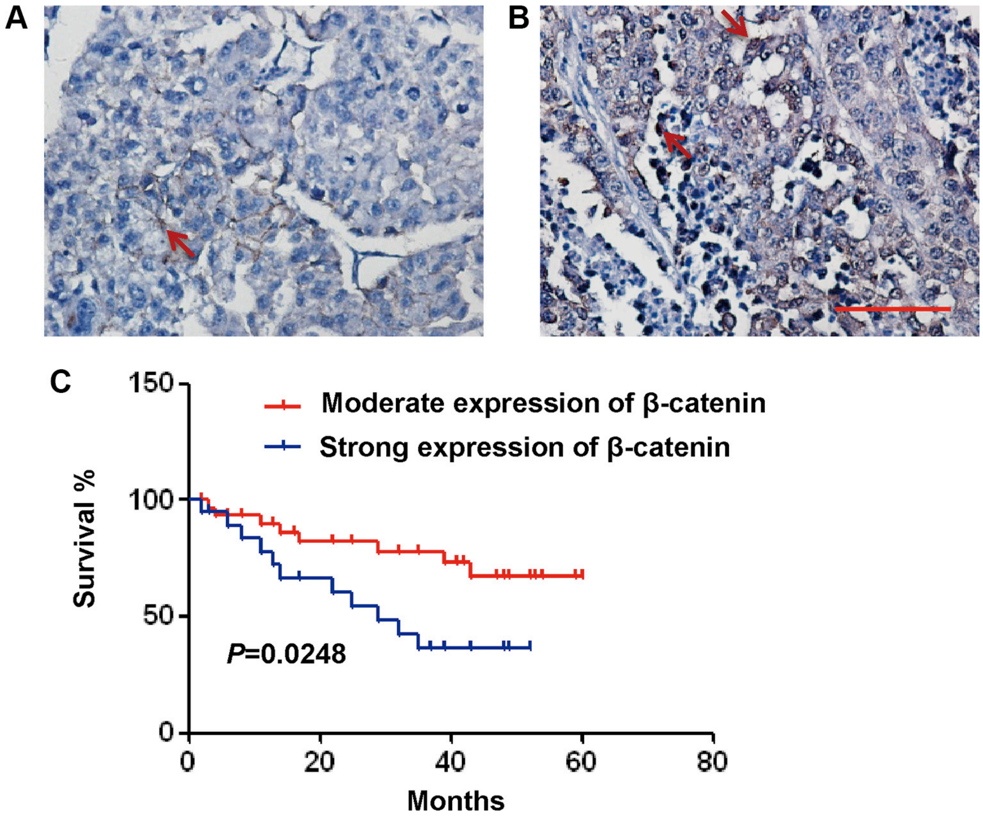

Supplementary Figure S4: (**A**) Moderate expression of β-catenin in HCC tissues. (Scale bar = 100 μm). (**B**) High expression levels of β-catenin in HCC tissues. (**C**) Kaplan-Meier polts illustrate that survival is based on the expression of β-catenin.
